# An Ethanol Extract Derived from *Bonnemaisonia hamifera* Scavenges Ultraviolet B (UVB) Radiation-Induced Reactive Oxygen Species and Attenuates UVB-Induced Cell Damage in Human Keratinocytes

**DOI:** 10.3390/md10122826

**Published:** 2012-12-14

**Authors:** Mei Jing Piao, Yu Jae Hyun, Suk Ju Cho, Hee Kyoung Kang, Eun Sook Yoo, Young Sang Koh, Nam Ho Lee, Mi Hee Ko, Jin Won Hyun

**Affiliations:** 1 School of Medicine, Jeju National University, Jeju 690-756, Korea; E-Mails: meijing0219@hotmail.com (M.J.P.); andyhave@naver.com (Y.J.H.); sukjucho@gmail.com (S.J.C.); pharmkhk@jejunu.ac.kr (H.K.K.); eunsyoo@jejunu.ac.kr (E.S.Y.); yskoh7@jejunu.ac.kr (Y.S.K.); 2 Department of Chemistry, College of Natural Sciences, Jeju National University, Jeju 690-756, Korea; E-Mail: namho@jejunu.ac.kr; 3 Jeju Biodiversity Research Institute, Jeju Technopark, Jeju 699-943, Korea; E-Mail: miheeko@jejutp.or.kr

**Keywords:** *Bonnemaisonia hamifera*, human keratinocytes, photoprotection, reactive oxygen species, ultraviolet B

## Abstract

The present study investigated the photoprotective properties of an ethanol extract derived from the red alga *Bonnemaisonia hamifera *against ultraviolet B (UVB)-induced cell damage in human HaCaT keratinocytes. The *Bonnemaisonia hamifera *ethanol extract (BHE) scavenged the superoxide anion generated by the xanthine/xanthine oxidase system and the hydroxyl radical generated by the Fenton reaction (FeSO_4_ + H_2_O_2_), both of which were detected by using electron spin resonance spectrometry. In addition, BHE exhibited scavenging activity against the 1,1-diphenyl-2-picrylhydrazyl radical and intracellular reactive oxygen species (ROS) that were induced by either hydrogen peroxide or UVB radiation. BHE reduced UVB-induced apoptosis, as shown by decreased apoptotic body formation and DNA fragmentation. BHE also attenuated DNA damage and the elevated levels of 8-isoprostane and protein carbonyls resulting from UVB-mediated oxidative stress. Furthermore, BHE absorbed electromagnetic radiation in the UVB range (280–320 nm). These results suggest that BHE protects human HaCaT keratinocytes against UVB-induced oxidative damage by scavenging ROS and absorbing UVB photons, thereby reducing injury to cellular components.

## 1. Introduction

Ultraviolet (UV) radiation comprises UVA, UVB and UVC rays and causes various health problems. UVB rays (280–320 nm) are particularly damaging to the basal cell layer of the epidermis. UVA and UVB rays stimulate the production of reactive oxygen species (ROS) in epidermal cells, resulting in skin lesions, accelerated aging [1] and the development of malignant skin diseases [2,3,4]. 

ROS are produced naturally within cells to facilitate biochemical processes, including mitochondrial electron transport. However, when ROS are produced in excess, cellular antioxidant defense mechanisms become overwhelmed, culminating in a cascade of ROS generation that eventually results in oxidative stress. For example, exposure of human keratinocytes to UVB radiation leads to the immediate generation of the superoxide anion [5]. The superoxide anion can then be converted into other ROS and free radicals, which have harmful effects on the skin, such as hydrogen peroxide and the hydroxyl radical [6]. 

The high concentrations of UVB-induced intracellular ROS mediate oxidative damage to cellular structures and biomolecules (*i.e.*, plasma membranes, lipids, proteins and nucleic acids). Therefore, ROS play a powerful role in the promotion of tumorigenesis [7,8]. Although excess ROS can be balanced by cellular antioxidant reactions, oxidative damage to the skin accumulates during ROS attacks, and ROS-mediated modification of biomolecules contributes to the development of many degenerative diseases and cancers [9]. It has been reported that UVA and UVB can alter both enzymatic and non-enzymatic antioxidants, and thus significantly affect the antioxidant defense of the various layers of skin and increase ROS level [10]. Other studies have demonstrated that accumulation of ROS within UVB-treated keratinocytes may promote programmed cell death, apoptosis [10,11,12].

In recent years, many studies have demonstrated the protective properties of marine algae-derived compounds against UV-induced cell damage. For instance, mycosporine-like amino acids synthesized by the red alga *Ptercladiella capillacea* inhibit UVB-induced lipid peroxidation [13], and the red alga *Porphyra yezoensis* has the source of UVB-absorbing substances that block thymine photodimer production [14]. In addition, an extract derived from the diatom *Phaeodactylum tricornutum *stimulates proteasome activity to increase intracellular protein turnover and the degradation of oxidized proteins following exposure to UVB radiation [15]. 

*Bonnemaisonia hamifera* (Bonnemaisoniales) is a red alga found in Europe, the Atlantic Islands, North America, the Caribbean Islands, Africa, and Asia [16]. In Korea, *B. hamifera* is widely cultivated on Jeju Island [17,18,19]. Previous studies report that aqueous extract of *B. hamifera *showed cytotoxic activity against B-16 murine melanoma cell line [20]. A poly-brominated 2-heptanone isolated from the *B. hamifera* inhibits bacterial colonization [21] and ethyl acetate extract of *B. hamifera *inhibits inflammation in mouse macrophage cell line RAW 264.7 [22]. Notably, *B. hamifera* extract also possesses antioxidant properties [23]. However, very little research has been conducted regarding the protective effects of *B. hamifera* against UVB radiation. Therefore, the aim of the present study was to examine the ability of a *B. hamifera* ethanol extract (BHE) to protect human HaCaT keratinocytes from UVB-induced cell damage.

## 2. Results and Discussion

### 2.1. Scavenging Effect of BHE against Free Radicals

**Figure 1:**
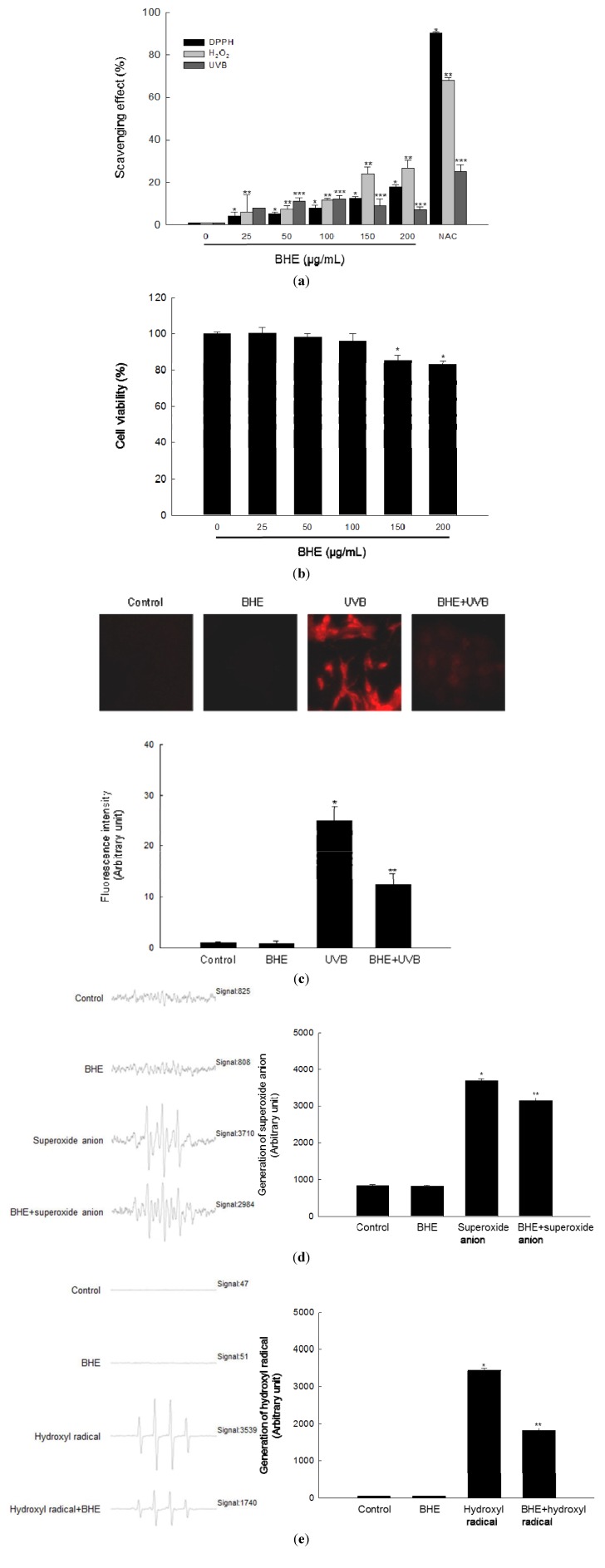
Scavenging effect of *Bonnemaisonia hamifera* ethanol extract (BHE) against free radicals. (**a**) Levels of the 1,1-diphenyl-2-picrylhydrazyl (DPPH) radical were measured spectrophotometrically at 520 nm. Intracellular reactive oxygen species (ROS) levels generated by H_2_O_2_ or ultraviolet B (UVB) radiation were detected using a spectrofluorometer after 2′,7′-dichlorodihydrofluorescein diacetate (DCF-DA) staining. BHE was used at the indicated concentrations. *N*-acetyl cysteine (NAC) served as the positive control. * Significantly different from the DPPH group, ** significantly different from the H_2_O_2_-treated group, and *** significantly different from the UVB-irradiated group (*p* < 0.05). (**b**) Cells were seeded, and BHE was added to a final concentration of 25, 50, 100, 150, or 200 μg/mL. After 24 h, cell viability was determined using the MTT assay. * Significantly different from control (*p *< 0.05). (**c**) Representative confocal images showing the increase in DCF red fluorescence intensity produced from DCF-DA by ROS in UVB-irradiated cells compared with that in control, BHE alone-treated, and BHE-pre-treated, UVB-irradiated cells. The fluorescence intensity was quantified. * Significantly different from control and ** significantly different from UVB-irradiated cells. (**d**) Superoxide anions generated by the xanthine/xanthine oxidase system were reacted with 5,5-dimethyl-1-pyrroline-*N*-oxide (DMPO) and the resultant DMPO/·OOH adducts were detected using ESR spectrometry. Representative peak data and a histogram for superoxide anion generation are shown. * Significantly different from control and ** significantly different from superoxide anion generated by the xanthine/xanthine oxidase system. (**e**) The hydroxyl radical generated by the Fenton reaction (H_2_O_2_ + FeSO_4_) was reacted with DMPO and the resultant DMPO/·OH adducts were detected by electron spin resonance (ESR) spectrometry. Representative peak data and histogram for hydroxyl radical generation are shown. * Significantly different from control and ** significantly different from hydroxyl radical generated by the Fenton reaction.

We first evaluated the ability of BHE to scavenge free radicals in a cell-free system. BHE scavenged the 1,1-diphenyl-2-picrylhydrazyl (DPPH) radical in a concentration-dependent manner. The extract scavenged 4% of the radical at a concentration of 25 μg/mL, 5% at 50 μg/mL, 8% at 100 μg/mL, 13% at 150 μg/mL, and 18% at 200 μg/mL, compared with 90% for 2 mM of *N*-acetyl cysteine (NAC), a well-known ROS scavenger that was used as the positive control (Figure 1a, black bars). 

We then evaluated the ability of BHE to scavenge intracellular ROS. BHE also scavenged H_2_O_2_-induced intracellular ROS in a concentration-dependent manner. The scavenging activity of BHE was 6% at 25 μg/mL, 7% at 50 μg/mL, 11% at 100 μg/mL, 24% at 150 μg/mL and 27% at 200 μg/mL, compared with 68% for NAC (Figure 1a, light gray bars). Finally, the scavenging activity of BHE against UVB-induced intracellular ROS was 8% at 25 μg/mL, 11% at 50 μg/mL, 12% at 100 μg/mL, 9% at 150 μg/mL, and 7% at 200 μg/mL, compared with 25% for NAC (Figure 1a, dark gray bars). 

BHE did not show any cytotoxicity against human HaCaT keratinocytes at 25, 50 or 100 μg/mL; however, the BHE was cytotoxic at concentrations above 150 μg/mL (Figure 1b). Based on these results, 100 μg/mL was chosen as the optimal concentration of BHE for further investigation. Confocal microscopy demonstrated that the red fluorescence intensity (arbitrary unit) of dichlorodihydrofluorescein generated by reaction of 2′,7′-dichlorodihydrofluorescein diacetate (DCF-DA) and ROS in UVB-irradiated cells was reduced when the irradiated cells were pre-treated with 100 μg/mL BHE (Figure 1c). The relative red fluorescence intensity values were 25 and 12 for the control and BHE alone, respectively. Finally, the scavenging effects of BHE against the superoxide anion and the hydroxyl radical were investigated by electron spin resonance (ESR) spectrometry after reaction with 5,5-dimethyl-1-pyrroline-*N*-oxide (DMPO). The superoxide anion signal in the xanthine/xanthine oxidase system was 3683 (mean value), but this signal decreased to 3142 (mean value) (scavenging effect of superoxide anion = 15%) after BHE treatment (Figure 1d). The corresponding signals for the control and BHE alone were 844 and 832 (mean value), respectively. BHE also reduced the hydroxyl radical signal produced by the Fenton reaction (from 3437 to 1824 (mean value); scavenging effect of hydroxyl radical = 47%; Figure 1e). By comparison, the signals for the control and BHE alone were 49 and 50 (mean value), respectively.

### 2.2. Effect of BHE against UVB-Induced Apoptosis

**Figure 2:**
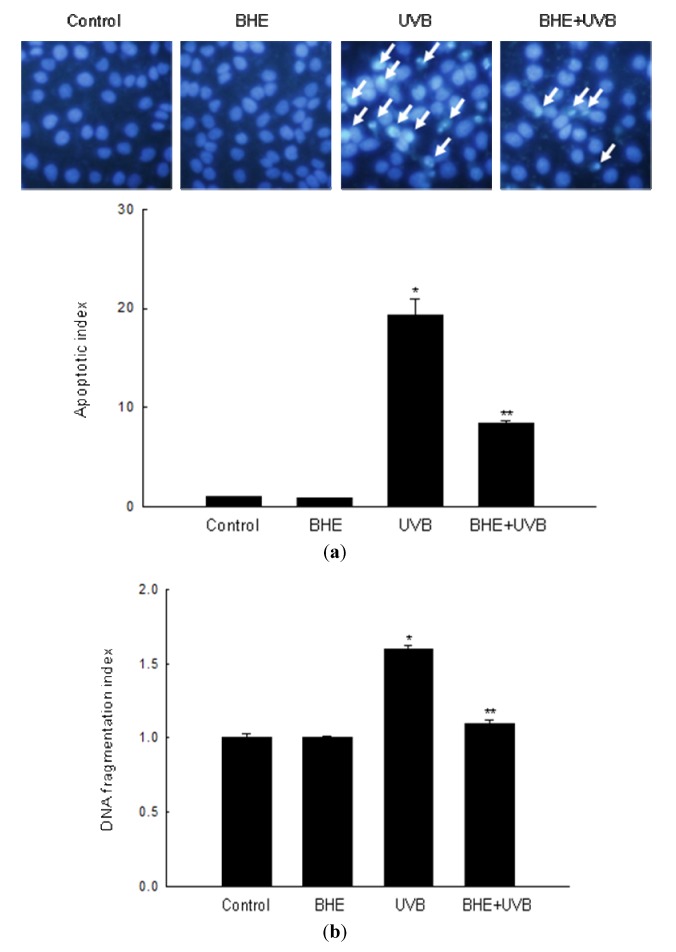
Protective effect of BHE against UVB-induced apoptosis. HaCaT cells were treated with BHE (100 μg/mL) and exposed to UVB radiation 1 h later. (**a**) Apoptotic bodies (arrows) were observed in cells stained with Hoechst 33342 dye by fluorescence microscopy and quantified. * Significantly different from control (*p *< 0.05) and ** significantly different from UVB-irradiated cells (*p *< 0.05). (**b**) Cytoplasmic histone-associated DNA fragmentation was quantified. * Significantly different from control (*p *< 0.05) and ** significantly different from UVB-irradiated cells (*p *< 0.05).

A number of recent studies show that UVB light induces apoptosis in keratinocytes [24,25,26]. In keeping with this observation, intact nuclei were observed in control, and cells treated only with BHE, whereas significant nuclear fragmentation (characteristic of apoptotic body formation) was observed in UVB-irradiated cells (apoptotic index, 19.3). However, nuclear fragmentation was markedly reduced in UVB-irradiated cells that were pre-treated with BHE (apoptotic index, 8.4) (Figure 2a). Similarly, the cytoplasmic histone-associated DNA fragmentation index decreased from 1.6 in UVB-irradiated cells to 1.1 in cells treated with BHE prior to UVB irradiation (Figure 2b). 

### 2.3. Effect of BHE against UVB-Induced Oxidative Lipid, Protein and DNA Damage

**Figure 3:**
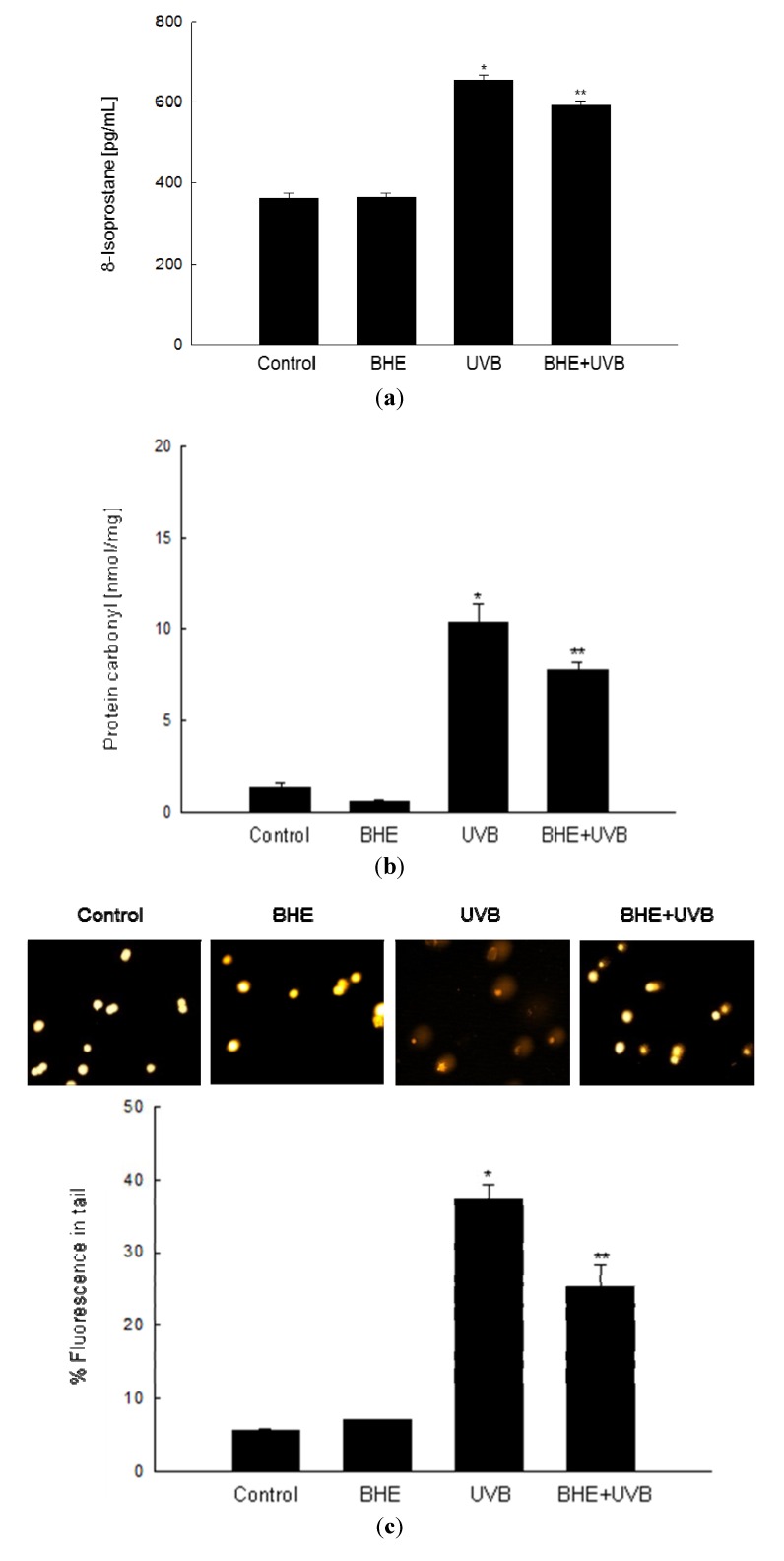
Protective effects of BHE against UVB-induced damage to cellular components. HaCaT cells were treated with BHE (100 μg/mL) for 1 h and then exposed to UVB radiation. (**a**) Following a 24 h incubation, lipid peroxidation was assayed by measuring the levels of 8-isoprostane secreted into the culture medium; (**b**) Protein oxidation was assayed by measuring the levels of carbonylated protein; (**c**) DNA damage was assessed by conducting an alkaline comet assay. Representative images and the percentage of total DNA fluorescence in the comet tails are shown. * Significantly different from control (*p *< 0.05) and ** significantly different from UVB-irradiated cells (*p *< 0.05).

Free radicals formed by UVB irradiation of cellular components may result in lipid peroxidation [27] and protein and DNA oxidation [28,29]. The ability of BHE to inhibit membrane lipid peroxidation, oxidative protein modification, and cellular DNA damage in UVB-irradiated HaCaT cells was investigated 24 h after the cells were exposed to UVB light. Lipid peroxidation was monitored by measuring the amount of 8-isoprostane secreted into the culture medium. As shown in Figure 3a, 8-isoprostane levels were significantly augmented in UVB-irradiated cells (mean level, 653 pg/mL) relative to control cells, whereas the response was dampened in UVB-irradiated cells that were pre-treated with BHE (mean level, 592 pg/mL). Protein carbonylation is a hallmark of oxidative stress-induced injury [30], and protein carbonyls accumulate in photodamaged skin [31]. The protein carbonyl content of UVB-irradiated HaCaT cells (mean level, 10.4 nmol/mg) increased significantly compared with that of control cells; however, the increase was attenuated in cells pre-treated with BHE (mean level, 7.8 nmol/mg) (Figure 3b). 

UVB-Induced damage to cellular DNA was detected in an alkaline comet assay. Exposure of cells to UVB rays increases the number of DNA breaks and, thus, the fluorescence intensity in the tails of the comet-like structures formed during the assay. As shown in Figure 3c, UVB-irradiated cells demonstrated notable comet formation, whereas BHE pre-treated, UVB-irradiated cells was decreased comet formation. The percentage of total DNA fluorescence in the comet tails of UVB-irradiated cells was 37%, while that in BHE-pre-treated, UVB-irradiated cells was 25%. The corresponding values for the untreated and BHE-treated controls were less than 10% (Figure 3c).

### 2.4. Effect of BHE on UVB Absorption

**Figure 4:**
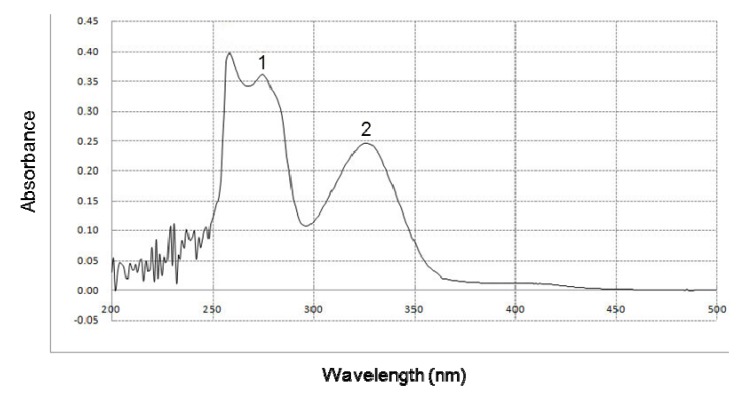
Effect of BHE on UVB absorption. UV/Visible light spectroscopic measurements were performed using a spectral range of 200–500 nm. Peaks 1 and 2 indicate the peak positions of absorbance at 275 and 326 nm, respectively.

The ability of BHE to absorb UVB rays was determined by UV/visible light spectrophotometry. BHE showed a high absorptive capacity in the range of UVB light (280–320 nm), with peak positions at 275 and 326 nm (Figure 4). 

### 2.5. Chromatography Pattern of BHE

**Figure 5:**
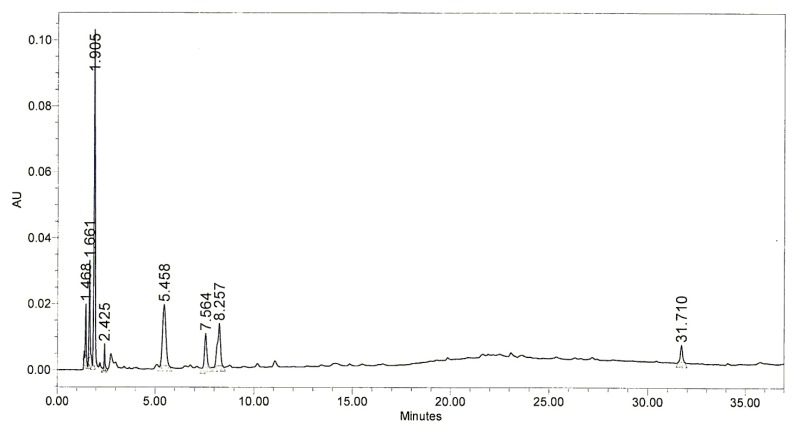
HPLC chromatogram of BHE. The retention time of each peak was shown.

A high-performance lipid chromatography (HPLC) was subsequently performed to determine which components of BHE might have been responsible for the photoprotective effect of BHE. HPLC profile of BHE was obtained from the Jeju Biodiversity Research Institute (Jeju, Korea). The HPLC profile demonstrated that BHE contained the main peaks at retention time of 1.905 min, 5.458 min, 7.564 min, and 8.257 min (Figure 5). Hence, determination of the compounds for these peaks responsible for the protective effects of BHE against UVB-induced damage should be conducted in further study.

### 2.6. Discussion

UV rays are one of the most relevant environmental factors affecting human quality of life, especially with regard to its hazardous health effects (e.g., premature skin aging, skin cancer, and exacerbation of infectious diseases) [32,33]. UVB rays have less penetrating power than UVA rays and act primarily on the epidermal basal layer of the skin. However, UVB radiation is more genotoxic and, therefore, capable of causing more extensive cell damage than UVA radiation. Many studies reported that UVB radiation induces ROS (e.g., singlet oxygen, hydrogen peroxide, and hydroxyl radical) in epidermal keratinocytes and leads to cell damage) [34,35,36]. Many of the antioxidant extracts or compounds from marine algae protected efficiently cells against UVB-induced oxidative stressed cell damage [37,38,39]. Our study demonstrated that red alga *B. hamifera *extract, BHE (100 μg/mL) scavenged 12% of UVB-induced intracellular ROS, as well as 11% of H_2_O_2_-induced ROS, the 15% of superoxide anion, the 47% of hydroxyl radical, and the 8% of DPPH radical; suggesting that BHE has effectively the quenching effect on hydroxyl radical. The antioxidant effect of BHE was also reported by Heo *et al.* (2006) [23]; *B. hamifera* methanol and water extract exhibited the scavenging activities against hydroxyl radical, superoxide anion, hydrogen peroxide, and DPPH free radicals [23]. There were reported that *B. hamifera* produces a large number of secondary metabolites, in particular halogenated compounds including 3-bromo-2-heptanoic acids and 3-bromo-2-nonanoic acids [40,41,42,43,44,45]. The halogenated compounds isolated from marine red alga exhibited various biological effects. For example, a halogenated phenols metabolite, 2,3,6-tribromo-4,5-dihydroxybenzyl methyl ether isolated from the red alga *Symphyocladia latiuscula *has shown antioxidant and antimicrobial activity [46]. And other halogenated compounds from marine alga exhibited antibacterial, antifungal, antiviral, anti-inflammatory, antiproliferative, cytotoxic, and insecticidal activity [47,48,49,50]. Also, some marine algae to protect themselves from UV rays have UV-absorbing substances that inhibit the penetration of UV ray into their tissue or cells. The major type of UV-absorbing substances found in red algae was identified as mycosporine-like amino acids having an absorption maximum of 300–350 nm and mainly absorbing UVA and UVB [14,51]. The red alga *Porphyra yezoensis* contained mycosporine-like amino acids such as palythine, shinorine, and porphyra-334 and showed UV absorbing effect [14,51]. In this study, BHE exhibited the absorptive capacity in the range of UVB light (280–320 nm). Thus, further study is needed to elucidate whether BHE may contain the mycosporine-like amino acids or other UV-absorbing substances. 

UVB exposure leads to the generation of ROS, causing damage to lipid membranes, accumulation of modified protein carbonyls, and DNA strand breaks [52,53,54]. All of these processes may disrupt cellular function and contribute to apoptosis. The present study showed that BHE protected cell membrane lipids from UVB-induced peroxidative damage and reduced the level of carbonylated proteins augmented by UVB rays. BHE also significantly prevented comet formation in UVB-irradiated keratinocytes. In the skin, a delicate balance is maintained between keratinocyte proliferation and cell death to ensure terminal differentiation and is coordinated throughout all layers of the human epidermis [55]. When this balance is disturbed by UVB radiation, the cells cannot repair the resulting cellular component damage including DNA, resulting in apoptotic cell damage. Therefore, the suppression of UVB-induced apoptosis in keratinocytes is beneficial for the prevention of photo-damage. In the present study, we also showed that UVB radiation increased apoptosis in HaCaT keratinocytes, and that BHE protected keratinocytes against UVB-induced cell death; decreased apoptotic body formation and DNA fragmentation. UV-induced apoptosis is a complex event involving different pathways. These include the activation of the tumor suppressor gene p53, the triggering of cell death receptors directly by UV or by autocrine release of death ligands, and the oxidative stress accompanied by mitochondrial changes and cytochrome c release [56]. The extrinsic apoptotic pathway through death receptors such as tumor necrosis factor receptor activates caspase cascade. And the intrinsic or mitochondrial pathway of apoptosis is regulated by the Bcl-2 family of proteins such as the anti-apoptotic protein (Bcl-2, Bcl-xl, Bcl-w) and the pro-apoptotic protein (Bax, Bak, Bid) [57]. Therefore, BHE may exert its protective actions against UVB-induced cell death by interfering with one or both of these pathways. Further work will be required to explore this possibility.

## 3. Experimental Section

### 3.1. Preparation of BHE

The *B. hamifera* specimen was collected on Jeju Island (Korea). Voucher specimen was deposited at the herbarium of the Jeju Biodiversity Research Institute (Jeju, Korea). The specimen was air dried, and the desiccated material (128 g) was extracted with 80% ethanol at room temperature for 24 h and then evaporated under a vacuum. The 27 g of extract was obtained (21% of yield). BHE was dissolved in dimethyl sulfoxide (DMSO) and final concentration of DMSO in control or BHE treatment did not exceed the 0.05%.

### 3.2. Reagents

The 1,1-diphenyl-2-picrylhydrazyl (DPPH) radical, *N*-acetyl cysteine (NAC), 5,5-dimethyl-1-pyrroline-*N*-oxide (DMPO), 2′,7′-dichlorodihydrofluorescein diacetate (DCF-DA) and Hoechst 33342 dye were purchased from Sigma Chemical Co. (St. Louis, MO, USA). All other chemicals and reagents were of analytical grade.

### 3.3. Cell Culture

Human keratinocytes (HaCaT cells) were obtained from the Amore Pacific Company (Gyeonggi-do, Korea). Cells were cultured in Dulbecco’s Modified Eagle’s Medium (DMEM) containing 10% heat-inactivated fetal calf serum, streptomycin (100 μg/mL) and penicillin (100 units/mL). Cells were maintained at 37 °C in an incubator with a humidified atmosphere of 5% CO_2_.

### 3.4. Detection of the DPPH Radical

BHE at a concentration of 25, 50, 100, 150, or 200 μg/mL was added to a solution of DPPH (1 × 10^−4^ M) in methanol. The resulting reaction mixture was shaken vigorously. After 3 h, the amount of unreacted DPPH was measured at 520 nm using a spectrophotometer. The DPPH radical scavenging activity (%) was calculated as [(optical density of DPPH radical) − (optical density of treated group)/(optical density of DPPH radical)] × 100.

### 3.5. Detection of Intracellular ROS

The DCF-DA method was used to detect intracellular ROS levels, generated by either H_2_O_2_ or UVB radiation, in HaCaT keratinocytes [58]. To detect ROS in H_2_O_2_-treated cells, the cells were first seeded at a density of 1.5 × 10^5^ cells/well. Sixteen hours after plating, the cells were treated with BHE at a concentration of 25, 50, 100, 150, or 200 μg/mL. After 30 min, H_2_O_2_ (1 mM) was added to the plate. Cells were incubated for an additional 30 min at 37 °C, and DCF-DA solution (25 μM) was then added. Ten minutes after the addition of DCF-DA, the red fluorescence of the 2′,7′-dichlorofluorescein (DCF) product was detected and quantified by using a PerkinElmer LS-5B spectrofluorometer (PerkinElmer, Waltham, MA, USA). The intracellular ROS scavenging activity (%) was calculated as [(optical density of H_2_O_2_ or UVB treatment) − (optical density of treated group)/(optical density of H_2_O_2_ or UVB treatment)] × 100.

To detect ROS in UVB-irradiated cells, the cells were treated with BHE as described above. After 1 h, the cells were exposed to UVB radiation at a dose of 30 mJ/cm^2^. The UVB source was a CL-1000M UV Crosslinker (UVP, Upland, CA, USA), which was used to deliver an energy spectrum of rays (280–320 nm). Cells were incubated for an additional 24 h at 37 °C followed by the addition of DCF-DA solution (25 μM). The fluorescent DCF product was detected as described above. 

Microscopic image analysis of intracellular ROS was achieved by seeding the cells on a coverslip-loaded 6-well plate at a density of 2 × 10^5^ cells/well. Sixteen hours after plating, the cells were treated with BHE (100 μg/mL). One hour after BHE treatment, the plate was irradiated with UVB. Twenty four hours later, DCF-DA (100 μM) was added to each well, and the cells were incubated for an additional 30 min at 37 °C. After washing with phosphate-buffered saline (PBS), the stained cells were mounted onto a microscope slide in mounting medium (Dako, Carpinteria, CA, USA). Images of the cells were collected and DCF fluorescence was quantified using a confocal microscope and the laser scanning microscope 5 PASCAL program (Carl Zeiss, Jena, Germany).

### 3.6. Cell Viability Assay

The effect of BHE on the viability of HaCaT cells was assessed as follows: Cells seeded on a 96-well plate at a density of 1 × 10^5^ cells/mL were treated 16 h later with 25, 50, 100, 150 or 200 μg BHE/mL. MTT stock solution (50 μL, 2 mg/mL) was added to each well to yield a total reaction volume of 200 μL. Four hours later, the plate was centrifuged at 800× *g* for 5 min and the supernatants were aspirated. The formazan crystals in each well were dissolved in DMSO (150 μL), and the absorbance at 540 nm was read on a scanning multi-well spectrophotometer [59].

### 3.7. Detection of the Superoxide Anion

The superoxide anion was produced via the xanthine/xanthine oxidase system and then reacted with a nitrone spin trap (DMPO). The DMPO/·OOH adducts were then detected using a JES-FA electron spin resonance (ESR) spectrometer (JEOL, Tokyo, Japan) [60,61]. Briefly, the ESR signaling was recorded 5 min after 20 μL of xanthine oxidase (0.25 U/mL) was mixed with 20 μL each of xanthine (10 mM), DMPO (3 M) and BHE (100 μg/mL). The ESR spectrometer parameters were: A magnetic field of 336 mT, power of 1.00 mW, frequency of 9.4380 GHz, modulation amplitude of 0.2 mT, gain of 500, scan time of 0.5 min, scan width of 10 mT, time constant of 0.03 s and a temperature of 25 °C. The value of superoxide anion generation was described as detected signal value (arbitrary unit).

### 3.8. Detection of the Hydroxyl Radical

The hydroxyl radical was generated by the Fenton reaction (H_2_O_2_ + FeSO_4_) and then reacted with DMPO. The resultant DMPO/·OH adducts were detected by ESR spectrometry [62,63]. The ESR spectrum was recorded 2.5 min after a phosphate buffer solution (pH 7.4) was mixed with 0.2 mL each of DMPO (0.3 M), FeSO_4_ (10 mM), H_2_O_2_ (10 mM), and BHE (100 μg/mL). The ESR spectrometer parameters were: A magnetic field of 336 mT; power of 1.00 mW; frequency of 9.4380 GHz; modulation amplitude of 0.2 mT; gain of 200; scan time of 0.5 min; scan width of 10 mT; time constant of 0.03 s; and a temperature of 25 °C. The value of hydroxyl radical generation was described as detected signal value (arbitrary unit).

### 3.9. Nuclear Staining with Hoechst 33342

Cells were treated with BHE (100 μg/mL) and exposed to UVB radiation 1 h later. Cells were incubated for an additional 24 h at 37 °C. Hoechst 33342 (10 mg/mL stock; 1.5 μL), a DNA-specific fluorescent dye, was added to each well and the cells were incubated for 10 min at 37 °C. The stained cells were visualized under a fluorescence microscope equipped with a CoolSNAP-Pro color digital camera (Media Cybernetics, Rockville, MD, USA). The degree of nuclear condensation was evaluated, and the number of apoptotic cells was quantified. Apoptotic index was calculated as (apoptotic cells in treated group/total cells in treated group)/(apoptotic cells in control group/total cells in control group).

### 3.10. DNA Fragmentation

Cellular DNA fragmentation was assessed by analyzing the extent of cytoplasmic histone-associated DNA fragmentation with a kit from Roche Diagnostics (Portland, OR, USA), according to the manufacturer’s instructions.

### 3.11. Lipid Peroxidation Assay

Cells were pretreated with 100 μg/mL BHE for 1 h and exposed to UVB, followed by incubation for an additional 24 h at 37 °C. Lipid peroxidation was assayed by determining the levels of 8-isoprostane secreted into the culture medium [64]. A commercial enzyme immunoassay (Cayman Chemical, Ann Arbor, MI, USA) was used according to the manufacturer’s instructions. 

### 3.12. Protein Carbonyl Formation

The extent of protein carbonyl formation was determined using an Oxiselect™ protein carbonyl ELISA kit (Cell Biolabs, San Diego, CA, USA), according to the manufacturer’s instructions.

### 3.13. Single-Cell Gel Electrophoresis (Comet Assay)

The extent of oxidative DNA damage was determined using the alkaline comet assay [65,66]. A suspension of HaCaT cells was mixed with 0.5% low melting agarose (LMA; 75 μL) at 39 °C, and the mixture was spread on a fully-frosted microscopic slide pre-coated with 1% normal melting agarose (NMA; 200 μL). After solidification of the agarose, the slide was covered with 0.5% LMA (75 μL) and then immersed in a lysis solution (2.5 M NaCl, 100 mM Na-EDTA, 10 mM Tris, 1% Trion X-100, and 10% dimethyl sulfoxide (DMSO), pH 10) for 1 h at 4 °C. The slides were then placed in a gel electrophoresis apparatus containing 300 mM NaOH and 10 mM Na-EDTA (pH 13) for 40 min to allow for DNA unwinding and the expression of the alkali-labile damage. An electrical field was then applied (300 mA, 25 V) for 20 min at 4 °C to draw the negatively charged DNA towards the anode. The slides were washed three times (for 5 min each time) at 4 °C in a neutralizing buffer (0.4 M Tris, pH 7.5), stained with propidium iodide (20 μg/mL, 75 μL) and observed under a fluorescence microscope equipped with a Komet 5.5 image analysis system (Kinetic Imaging, UK). The percentage of total DNA fluorescence in the comet tails and the tail lengths were recorded for 50 cells per slide.

### 3.14. UV/Visible Light Absorption Analysis

To study the UVB absorption spectra of BHE, the extract was diluted in DMSO at a ratio 1:500 (v/v) and the solution was then scanned with UV light (200–500 nm) using a Biochrom Libra S22 UV/visible light spectrophotometer (Biochrom Ltd., Cambridge, UK).

### 3.15. HPLC Analysis

BHE was dissolved in 10% acetonitrile at 1% concentration and filtered through 0.2 μm syringe filter. The HPLC system consisted of Waters (Waters Corporation, MA, USA) Alliance equipped with a Waters e2695 separation module and a Waters 2998 PDA detector. Chromatographic separation was performed using a Waters, Sunfire C-18 (5 μm, 4.6 mm × 150 mm) column and monitored at 270 nm by PDA. The solvents were mixture of aqueous 0.5% acetic acid and acetonitrile. The HPLC system was operated at a flow rate of 1 mL/min and the injection volume 10 μL with a column temperature at 25 °C. 

### 3.16. Statistical Analysis

All measurements were performed in triplicate, and all values are expressed as the mean ± the standard error. The results were subjected to an analysis of variance (ANOVA) followed by Tukey’s test to analyze differences between conditions. In each case, a *P* value of < 0.05 was considered statistically significant.

## 4. Conclusions

In conclusion, this study provides an initial demonstration of the potential applicability of BHE as a photoprotective agent for the skin. Further study will be directed towards elucidating the chemical composition of BHE and its mechanism of action.
